# Privacy-Preserving Image Classification Using ConvMixer with Adaptative Permutation Matrix and Block-Wise Scrambled Image Encryption

**DOI:** 10.3390/jimaging9040085

**Published:** 2023-04-18

**Authors:** Zheng Qi, AprilPyone MaungMaung, Hitoshi Kiya

**Affiliations:** Department of Computer Science, Tokyo Metropolitan University, 6-6 Asahigaoka, Hino-shi, Tokyo 191-0065, Japan; qizheng97@outlook.com (Z.Q.);

**Keywords:** privacy-preserving, ConvMixer, image encryption

## Abstract

In this paper, we propose a privacy-preserving image classification method using block-wise scrambled images and a modified ConvMixer. Conventional block-wise scrambled encryption methods usually need the combined use of an adaptation network and a classifier to reduce the influence of image encryption. However, we point out that it is problematic to utilize large-size images with conventional methods using an adaptation network because of the significant increment in computation cost. Thus, we propose a novel privacy-preserving method that allows us not only to apply block-wise scrambled images to ConvMixer for both training and testing without an adaptation network, but also to provide a high classification accuracy and strong robustness against attack methods. Furthermore, we also evaluate the computation cost of state-of-the-art privacy-preserving DNNs to confirm that our proposed method requires fewer computational resources. In an experiment, we evaluated the classification performance of the proposed method on CIFAR-10 and ImageNet compared with other methods and the robustness against various ciphertext-only-attacks.

## 1. Introduction

The spread of deep neural networks (DNNs) [1] has immensely contributed to solving complicated tasks for many applications. Training a DNN with a high generalization capability usually requires processing a large amount of data. Recently, it has been prevalent for data owners to utilize cloud servers to compute and process data because they provide a convenient platform and powerful machines for computing. Generally, data contains personally identifiable private information, and therefore, data privacy may be compromised in cloud environments. Privacy-sensitive datasets, such as of human faces and medical images, may be illegally accessed by a third party. Violation of data privacy raises legal issues such as the Health Insurance Portability and Accountability Act (HIPAA) [2] and General Data Protection Regulation (GDPR) [3]. Therefore, organizations like hospitals are not able to train DNN models in the cloud environments although deep learning has shown remarkable performance. Accordingly, it is crucial to protect data privacy in cloud environments, so privacy-preserving DNNs have become an urgent challenge [4,5].

To train/test DNN models in the cloud environment while preserving privacy, researchers have proposed numerous methods. Traditional cryptographic methods such as homomorphic encryption [6] may contribute to solving the problem, but the computation and memory costs are expensive, and it is not easy to apply these methods to DNNs directly. Federated learning [7] allows users to train a global model without centralizing the training data on one machine, but it cannot protect privacy during inference for test data when a model is deployed in an untrusted cloud server.

To overcome the above limitations, researchers have also proposed image encoding methods in a private way to protect privacy, although privacy guarantees are not as strong as cryptographic methods. Image encoding methods focus on protecting data privacy by encrypting plain data to visually protected data before uploading it to the cloud environment [8]. Such methods for privacy-preserving image classification [9], such as the GAN-based method, achieve a high classification accuracy, but they are not robust against some attacks [10]. On the other hand, block-wise scrambled images have been confirmed to be robust against various attacks, but it is difficult to avoid the influence of image encryption [11,12]. One of the solutions is to use a classification network with an adaptation network [13,14]. However, the adaptation network used for reducing the influence of encryption also increases the computation costs by a large amount, so images large in size cannot be applied to the adaptation network.

Therefore, we propose the combined use of a novel block-wise encryption method and a ConvMixer with an adaptive permutation matrix. A part of this work was presented in [15]. In this paper, we have added experiment results on the ImageNet dataset which was never applied to any leanable image encryption method before. We have also added security evaluation results and key space analysis to further confirm the effectiveness of the proposed method. In addition, we calculate the number of parameters and floating operations (FLOPs) to make a comparison between all state-of-the-art privacy-preserving DNNs. In an experiment, the proposed method is confirmed to maintain a satisfactory classification performance on both CIFAR-10 [16] and ImageNet [17] with fewer computation costs and strong robustness against various attack methods.

The rest of this paper is structured as follows. Section 2 presents materials and methods including the proposed method in details. Section 3 puts forward experiments and results. Discussion is presented in Section 4, and Section 5 concludes this paper.

## 2. Materials and Methods

### 2.1. Related Work

Generally, privacy-preserving machine learning considers privacy in the whole machine learning pipeline, i.e., the (1) privacy of datasets, (2) privacy of models, and (3) privacy of models’ outputs [6]. To address privacy, there are various methods such as cryptographic methods [18,19,20], federated learning [7,21,22], differential privacy [23,24,25], image encoding methods [13,14,26,27,28]. As we focus on the privacy of datasets for image classification tasks, we review learnable image encryption, image encoding methods, and isotropic networks that can be used to classify visually protected images in the following subsections.

#### 2.1.1. Learnable Image Encryption

Learnable image encryption is encryption that protects visual information of plain images without compromising the classification ability of deep neural networks. Tanaka first introduced a block-wise learnable image encryption method (LE) with an adaptation layer [13], which is used prior to a classifier to reduce the influence of image encryption. Another encryption method is a pixel-wise encryption (PE) method in which negative-positive transformation (NP) and color component shuffling are applied without using any adaptation layer [26]. However, both encryption methods are not robust enough against ciphertext-only attacks, as reported in [10,29]. To enhance the security of encryption, LE was extended to ELE by adding a block scrambling step and a pixel encryption operation with multiple keys [14]. However, ELE still has an inferior accuracy compared with using plain images, although an additional adaptation network (denoted as ELE-AdaptNet hereinafter) is applied to reduce the influence of the encryption. Moreover, images large in size cannot be applied to ELE because of the high computation cost of ELE-AdaptNet.

#### 2.1.2. Image Encoding Approaches

Image encoding approaches are privacy-preserving methods that encode images to hide visual information and are close to our proposed method. One method trains a U-Net with a pre-trained classifier as a transformation network to encode images, but this method can not protect the visual information in a training process [10]. Another method called InstaHide encodes images by mixing them with other images and applying a pixel-wise sign-flipping mask [27]. However, it has been proved that visual information can be reconstructed from the encoded images by an attack method in [30]. Recently, random neural network methods, such as NeuraCrypt [28], have been proposed with Vision Transformer (ViT) [31] to encode images, but the security of this method is risky since the encoded images and plain images can be matched correctly by an algorithm in [32].

#### 2.1.3. Isotropic Networks

Recently, isotropic networks with an embedding structure, such as ViT [31] and ConvMixer [33], have attracted more interest in computer vision tasks. The embeddings in isotropic networks have a structure equivalent to adaptation networks, so isotropic networks could be used as a classifier of block-wise scrambled images to reduce the influence of encryption without an adaptation network. A novel block-wise encryption was proposed that consists of block scrambling and simplified pixel shuffling with ViT (denoted as ViT-Enc) [34] and achieves a high classification performance, but it is not robust against attacks, as reported in [35]. Furthermore, isotropic networks are demonstrated to have a good classification performance with Encryption-then-Compression (EtC) images, as reported in [11]. Accordingly, we propose a novel privacy-preserving classification method with ConvMixer to optimize ELE and its adaptation network to reduce the computation cost and make it adapt to large images.

### 2.2. Overview

**Figure 1:**
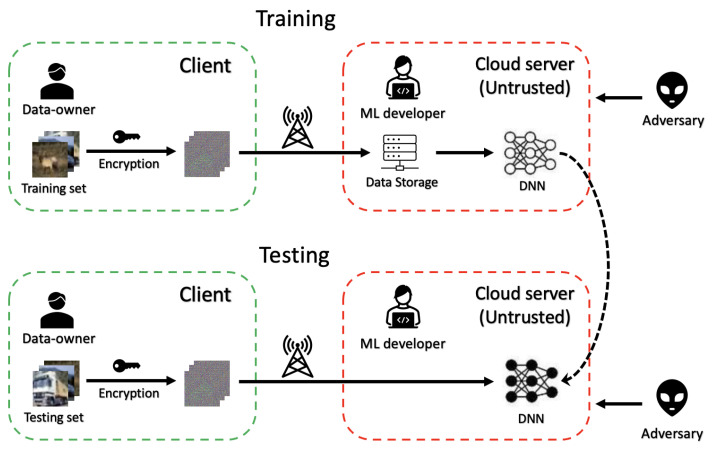
Scenario of proposed method.

To protect data privacy in cloud environments, we propose a privacy-preserving image classification method using block-wise encrypted images and a ConvMixer model with an adaptive permutation matrix. Figure 1 illustrates an overview of the scenario of the proposed method, in which we consider there to be three indispensable participants: a data owner, a machine learning (ML) developer, and an adversary.

**Data owner** requests the ML developer to train a model on a dataset with sensitive information on a cloud server, but he distrusts the cloud environment because an adversary may access his dataset and compromise the data privacy. Thus, he encrypts all the images (for both training and testing) in the dataset using the proposed encryption algorithm with a secret key before transmitting them to the ML developer. Note that only the data owner has the secret key and the unencrypted dataset.

**ML developer** provides the service that trains models for data owners on their cloud server. Since the cloud environment is not trusted generally, he receives only the encrypted images from the data owner. Images encrypted by the proposed encryption algorithm can be applied to DNNs directly, so he uses the encrypted images received from the data owner to train a model. After the training, the data owner can also use the encrypted images to test the model.

**Adversary** is an attacker or hacker who can access the cloud environment provided by the ML developer illegally and targets sensitive information in uploaded datasets. The proposed encryption algorithm conceals the perceptual information of plain images, so he cannot view any effective information from the encrypted images. Data privacy is preserved in this process. However, he still attempts to reconstruct the perceptual information from the encrypted images despite the lack of the key.

### 2.3. Threat Model

As seen in Figure 1, an adversary can only obtain only the encrypted dataset (without any perceptual information or key) if he accesses the cloud environments. However, it is difficult to disguise some apparent information, such as overall dataset information (image size and distribution) and the scheme of the proposed encryption. Thus, an adversary may perform ciphertext-only (COA) attacks via this information to restore the perceptual information from encrypted images.

### 2.4. Requirements

We aim to satisfy the following three requirements in consideration of the scenario of the proposed method and threat model.

1. Security: Any perceptual information of plain images should not be reconstructed from images encrypted by the proposed method unless the key is exposed. The proposed method is required to be robust against all ciphertext-only-attacks.
2. Model capability: Privacy-preserving methods for DNNs should not decrease the model capability severely. A classifier trained with images encrypted by the proposed method is required to maintain an approximate accuracy as when using plain images.
3. Computational requirement: Privacy-preserving DNNs should not increase the computational requirement in quantity. Training or testing a classifier with the proposed method is required to consume a similar amount of computational resources as standard classifiers.

### 2.5. Image Encryption Method

The proposed encryption method considers the property of the patch embedding structure in ConvMixer where the patch size is M×M. The procedure of the proposed method is as follows.

1. Divide an 8-bit RGB image into blocks with a block size of M×M.
2. Permutate the divided blocks randomly with a secret key $K_{1}$.
3. Perform pixel shuffling in every block with a secret key $K_{2}$, where $K_{2}$ is commonly used in all blocks.
4. Apply negative-positive transformation to each pixel in each block by using a secret key $K_{3}$, where $K_{3}$ is commonly used in all blocks.
5. Concatenate all the blocks to produce an encrypted image.

**Figure 2:**
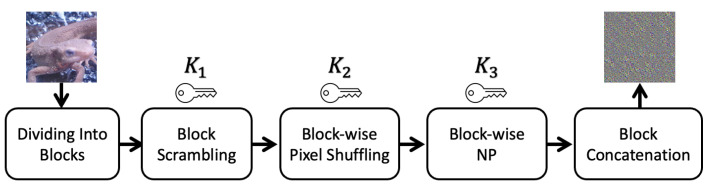
Pipeline of proposed encryption method.

Figure 2 depicts the pipeline of the proposed block-wise encryption method. We define block scrambling, pixel shuffling, and NP transformation as follows.

#### 2.5.1. Block Scrambling

1. An 8-bit RGB image is divided into blocks with a size of M×M as
  (1) $$B=\left\{B_{1},\ldots ,B_{i},\ldots ,B_{N}\right\},i\in \left\{1,\ldots ,N\right\}$$
  where *N* is the number of blocks, and $B_{i}$ is a divided block.
2. Generate a random permutation vector (secret key) $K_{1}$ as
  (2) $$K_{1}=\left[\alpha_{1},\ldots ,\alpha_{i},\ldots ,\alpha_{i^{\prime }},\ldots ,\alpha_{N}\right],i\in \left\{1,\ldots ,N\right\}$$
  where $\alpha_{i}\in \left\{1,\ldots ,N\right\}$ and $\alpha_{i}\neq \alpha_{i^{\prime }}$ if $i\neq i^{\prime }$.
3. Permute the blocks in *B* with $K_{1}$ such that $B_{i}^{\prime }=B_{\alpha_{i}}$ and permuted blocks are given by
  (3) $$B^{\prime }=\left\{B_{1}^{\prime },\ldots ,B_{i}^{\prime },\ldots ,B_{N}^{\prime }\right\},i\in \left\{1,\ldots ,N\right\}$$

#### 2.5.2. Block-Wise Pixel Shuffling

Assume that the image has been divided into blocks (dimension of 3×M×M) as

(4) $$B=\left\{B_{1},\ldots ,B_{i},\ldots ,B_{N}\right\},i\in \left\{1,\ldots ,N\right\}$$

where *N* is the number of blocks, and $B_{i}$ is a divided block.

1. Generate a random permutation vector $K_{2}$ as
  (5) $$K_{2}=\left[\beta_{1},\ldots ,\beta_{j},\ldots ,\beta_{j^{\prime }},\ldots ,\beta_{3M^{2}}\right],j\in \left\{1,\ldots ,3M^{2}\right\}$$
  where $\beta_{j}\in \left\{1,\ldots ,3M^{2}\right\}$ and $\beta_{j}\neq \beta_{j^{\prime }}$ if $j\neq j^{\prime }$.
2. For each block $B_{i}\in B$, repeat step 3–5.
3. Flatten three channels of each pixel in $B_{i}$ as
  (6) $$P=\left\{p_{1},\ldots ,p_{j},\ldots ,p_{3M^{2}}\right\},j\in \left\{1,\ldots ,3M^{2}\right\}$$
4. Permute the elements in *P* with $K_{2}$ such that $p_{j}^{\prime }=p_{\beta_{j}}$ and permuted elements are given by
  (7) $$P^{\prime }=\left\{p_{1}^{\prime },\ldots ,p_{j}^{\prime },\ldots ,p_{3M^{2}}^{\prime }\right\},j\in \left\{1,\ldots ,3M^{2}\right\}$$
5. Resize the vector $P^{\prime }$ to the original dimension (3×M×M).

#### 2.5.3. Block-Wise Negative Positive Transformation

Assume that the image has been divided into blocks (dimension of 3×M×M) as

(8) $$B=\left\{B_{1},\ldots ,B_{i},\ldots ,B_{N}\right\},i\in \left\{1,\ldots ,N\right\}$$

where *N* is the number of blocks, and $B_{i}$ is a divided block.

1. Generate a set of random binary numbers independently as
  (9) $$r_{k}=\left\{0,1\right\},k\in R^{3\times M\times M},r_{k}\in K_{3}$$
  where $r_{k}$ is distributed with 50% of “0”s and 50% of “1”s.
2. For each block $B_{i}\in B$, repeat step 3.
3. For each element $p_{k}$ in $B_{i}$, a transformed value is calculated by
  (10) $$p_{k}^{\prime }=\left\{\begin{array}{cc} p_{k} & r_{k}=0\\ p_{k}⊕2^{L} & r_{k}=1 \end{array}\right.,k\in R^{3\times M\times M}$$
  where *L* denotes the number of bits of an input image (L=8 in this paper).

### 2.6. ConvMixer with Adaptive Permutation Matrix

Conventional methods such as ELE append an adaptation network to a classifier, where ELE-AdaptNet consists of block-wise sub-networks, an adaptative permutation matrix, and a pixel shuffling layer. ELE-AdaptNet can reduce the influence of block-wise encryption while increasing the computation cost of the model.

**Figure 3:**
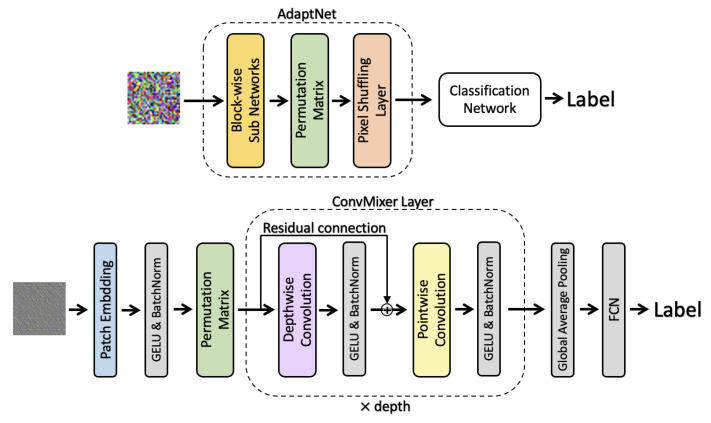
Framework of proposed ConvMixer and ELE-AdaptNet.

ConvMixer and ELE-AdaptNet share a similar architecture, so we propose only appending the adaptative permutation matrix to ConvMixer. Figure 3 shows the framework of the proposed ConvMixer compared with ELE-AdaptNet, in which an adaptative permutation matrix is added after patch embedding, and a resulting embedding is then used as an input to ConvMixer layers. The loss function used for the proposed method is given by

(11) $$L=L_{CE}+\lambda L_{U},$$

where $L_{CE}$ is the cross-entropy loss, $L_{U}$ is the penalty for the adaptive permutation matrix introduced in [14], and λ is a hyperparameter.

The proposed ConvMixer has two properties:

1. Block-wise sub-networks in ELE-AdaptNet aim to adapt to block-wise transformation, such as block-wise pixel shuffling with different keys. The patch embedding structure in ConvMixer enables us to reduce the influence of block-wise encryption without block-wise sub-networks.
2. An adaptative permutation matrix is designed to be trained as an inverse process of block scrambling so that the proposed ConvMixer can reduce the influence of block scrambling.

Therefore, the proposed ConvMixer does not need a whole ELE-AdaptNet but is still expected to reduce the influence of block-wise encryption.

### 2.7. Key Space

The key space describes a set of all possible permutations in an encryption algorithm. As seen in Figure 2, the proposed encryption algorithm consists of block scrambling, block-wise pixel shuffling, and NP transformation. For the case where an image is divided into blocks with a size of 3×M×M and the number of blocks in an image is *N*, the key space of each operation is calculated as follows.

(12) $$S_{bs}=N!$$

(13) $$S_{ps}=\left(3M^{2}\right)!$$

(14) $$S_{NP}=2^{3M^{2}}$$

Thus, the key space of the proposed method is calculated as

(15) $$S_{proposed}=S_{bs}\cdot S_{ps}\cdot S_{NP}=N!\cdot \left(3M^{2}\right)!\cdot 2^{3M^{2}}$$

When a 3×224×224-sized image is divided into blocks with a size of 3×16×16, the number of blocks is 196. The key space of the proposed method is

(16) $$S_{proposed}=196!\times \left(3\times 16\times 16\right)!\times 2^{3\times 16\times 16}≳2^{8242}$$

Therefore, the proposed encryption method provides a sizeable key space that enhances the robustness against various attacks.

### 2.8. Robustness against Ciphertext-Only Attacks

Recently, numerous ciphertext-only attack methods have been proposed to restore perceptual information from block-wise encrypted images. The jigsaw puzzle solver attack [36,37] attempts to decrypt block-scrambled images. However, assembling encrypted images was difficult if the number of blocks is large, the block size is small, and encrypted images have compression distortion and less color information [37]. Recently, the attack in [35] extends this attack to reverse the encryption process of ViT-Enc using edge information [34]. To prevent from this kind of attack, we apply full pixel shuffling in each block of the proposed encryption unlike ViT-Enc.

Furthermore, the feature reconstruction attack (FR-Attack) exploits local properties to refigure shapes from encrypted images [29]. This attack method is devised to break the specific encryption algorithms, so they are feeble against other encryption methods, including the proposed method. In addition, DNN-based ciphertext-only attacks are also very effective in some block-wise encryption methods. The generative adversarial network-based attack (GAN-attack) enables an adversary to train a GAN with a synthetic dataset and encrypted images to decrypt images [38]. An adversary may also perform an inverse transformation network attack (ITN-attack) if they are familiar with the encryption scheme [10]. The transformation model is trained by exact pairs of plain and encrypted images with random keys. Encryption methods that do not disturb spatial information, such as LE [13] and PE [26], are not robust against DNN-based attacks, but the block scrambling step in our proposed method hides an enormous amount of spatial information. The proposed method will be demonstrated to be robust against these attacks in Section 3.3.

## 3. Results

In this section, we performed a series of experiments to verify the effectiveness of the proposed method.

### 3.1. Details of Experiments

We conducted image classification experiments on the CIFAR-10 dataset [16] and the ImageNet dataset [17]. CIFAR-10 consists of 60,000 color images (with a dimension of 3×32×32) with 10 classes (6000 images for each class) where 50,000 images are for training and 10,000 for testing. ImageNet comprises 1.28 million color images for training and 50,000 color images for validation. We resized all images to a dimension of 224×224 for the proposed encryption.

We used the timm training framework as in the original ConvMixer paper (https://github.com/locuslab/convmixer accessed on 22 March 2023). The configurations of ConvMixer for CIFAR-10 were: a kernel size of 9, a depth of 16, and a hidden size of 512. The patch size of ConvMixer was always the same as the block-size in the proposed encryption. We used the training settings from [33] except for the training epochs. We trained ConvMixer models for 300 epochs for plain images and 400 epochs for encrypted images. In addition, hyperparameter λ in the loss function was set to 0.0001.

For ImageNet experiments, we fine-tuned the pretrained models with publicly available training code (https://github.com/webdataset/webdataset-lightning accessed on 22 March 2023). We chose a larger ConvMixer to evaluate our proposed encryption on ImageNet. The configurations of ConvMixer for ImageNet were: a patch size of 14, a kernel size of 9, a depth of 20, and a hidden size of 1024. The block-size in the encryption was still the same as the patch size. For plain images, we followed the same settings from [33]. For encrypted images, all layers except the adaptive permutation matrix were pre-trained on plain ImageNet, and we trained the adaptive permutation matrix from scratch. We used a learning rate of 0.01 to fine-tune the proposed ConvMixer for 15 epochs.

### 3.2. Classification Accuracy

#### 3.2.1. CIFAR-10

Table 1 shows the image classification performance and computation cost of the proposed method compared with state-of-the-art methods. The ConvMixer model with an adaptive permutation matrix achieved a satisfactory classification accuracy for images encrypted by the proposed encryption method with relatively less computation. In addition, without the adaptive permutation matrix, the accuracy of the ConvMixer model decreased by approximately 3%, and the use of the permutation matrix did not increase the computation cost by too much.

#### 3.2.2. ImageNet

The previous learnable encryption methods were never applied to the ImageNet dataset, so that it is difficult to train the previous methods on the ImageNet dataset. Therefore, we were unable to directly make a comparison on ImageNet. However, the proposed method can be applied to the ImageNet dataset by taking advantage of pre-trained models. Table 2 shows the accuracy of both plain and encrypted images. Our proposed method achieved a 63.72% accuracy on ImageNet, so the proposed method can adapt to various scales of datasets.

### 3.3. Robustness against Attacks

**Figure 4:**
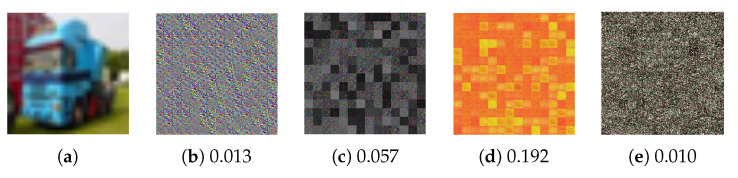
Example of images restored from ones encrypted by ConvMixer-Encryption. (**a**) Plain, (**b**) Encrypted, (**c**) FR-attack, (**d**) GAN-attack, (**e**) ITN-attack.

We conducted the FR-Attack [29], GAN-Attack [38], and ITN-Attack [10] to confirm the robustness of the proposed encryption method on the CIFAR-10 dataset. We followed almost the same settings as in their original papers except for some modifications to make these attack methods fit the image size of 3×224×224 used for the proposed method. Figure 4 shows images restored by using the three attacks. Structural similarity index measure (SSIM) [39] values are marked at the bottom of the restored images to illustrate the structural similarity between a restored image and a plain one. A larger value means a higher structural similarity between the two images. The results from Figure 4 demonstrated that the perceptual information of plain images could not be reconstructed by these attack methods, so the proposed method was robust against ciphertext-only attacks.

## 4. Discussion

**Figure 5:**
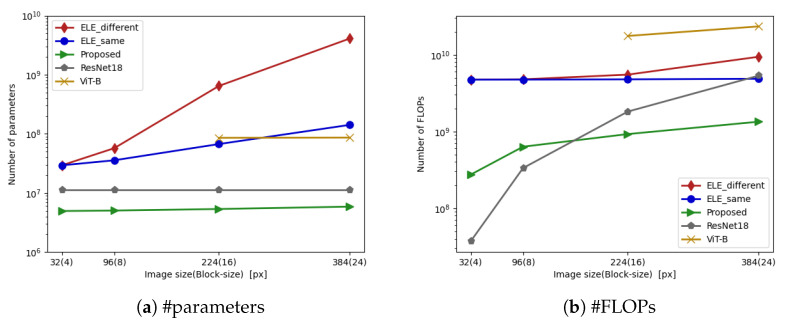
Number of parameters and FLOPs in privacy-preserving DNNs. Both “ELE_same” and “ELE_different” consist of ELE-AdaptNet and Shakedrop network.

In this section, we first discuss the computation cost in terms of the number of parameters and FLOPs for well-known privacy-preserving DNNs under different image sizes, and overall evaluation. We formulate the number of parameters in ELE-AdaptNet and the proposed ConvMixer in accordance with their architecture. Figure 5 shows a graph of the number of parameters and FLOPs versus image sizes. The number of parameters in ELE-AdaptNet with its classifier and the proposed method is calculated by Equations (17) and (19). The number of FLOPs is estimated with this code (https://github.com/facebookresearch/fvcore accessed on 22 March 2023).

### 4.1. Classifier with Adaptation Network

Conventional methods such as ELE need the combined use of an adaptation network and a classifier for improving the classification performance (see Figure 3). In the adaptation network, sub-networks transform each block using a convolutional layer (with $3\times output\_channel\times kernel\_size^{2}$ parameters) and a BatchNorm2d (with 2×output_channel parameters) separately, and then the results are integrated and multiplied by a permutation matrix (n×n parameters).

Let output_channel (hidden size) be *h* and kernel_size be *k*. When an 8-bit RGB image is segmented into blocks with a block size of *M*, there are *n* blocks in an image. Note that the sub-networks in the adaptation network are intended to reduce the influence of encryption, so kernel_size and block size *M* are the same. The total number of trainable parameters in the ELE-AdaptNet is given as

(17) $$\begin{array}{cc} N_{ELE} & =N_{AdaptNet}+N_{classifier}\\ & =N_{sub-networks}+N_{matrix}+N_{classifier}\\ & =n\left(3\cdot h\cdot M^{2}+2\cdot h\right)+n^{2}+N_{classifier}. \end{array}$$

Since the Shakedrop network [40] has never been trained or tested on a large image, we do not consider the computational growth of the classifier for ELE in this research. For the adaptation network of ELE, when the size of input images becomes larger, using the same hidden size *h* (denoted as ELE_same) for convolutional layers in the sub-networks will lead an output representation with a smaller number of channels. This might degrade the performance of the classifier. Using a larger hidden size *h* (denoted as ELE_different) can increase the number of channels in the output representation but also increase the number of parameters and FLOPs in the adaptation network drastically. All in all, the combined use of ELE-AdaptNet and a classifier for ELE images generates too much growth in computation cost, especially for large images. In addition, it is noteworthy that a heavier adaptation network relative to the classifier might make the training more difficult.

### 4.2. ConvMixer with Adaptive Permutation Matrix

Unlike the ELE, the proposed method adds a permutation matrix only to ConvMixer. The number of parameters in ConvMixer is given as in the original paper,

(18) $$N_{ConvMixer}=h\left[d\left(k^{2}+h+6\right)+3M^{2}+n_{\mathrm{classes}}+3\right]+n_{\mathrm{classes}},$$

where *h* is hidden size, *d* is depth, *k* is kernel size, and $n_{\mathrm{classes}}$ is number of classes. Note that we use the block size *M* as a patch size in ConvMixer. The total number of parameters for the modified ConvMixer is given as

(19) $$\begin{array}{cc} N_{Proposed} & =N_{ConvMixer}+N_{matrix}\\ & =N_{ConvMixer}+n^{2}. \end{array}$$

As shown in to Figure 5, the proposed method does not increase the number of parameters and FLOPs significantly even when large image sizes are used, and it has a relatively small amount of computation compared with other privacy-preserving DNNs in most cases.

### 4.3. Other Privacy-Preserving DNNs

Unmodified ResNet18 [41] and ViT-B are used as classifiers for PE and ViT-Enc, respectively, because these encryption algorithms are designed with adaptability to classifiers, so neither of them has an extra computation cost when using encrypted images. Using larger images for ViT and ResNet18 models increases the number of FLOPs but maintains a similar number of parameters. For the ViT-B model, smaller images are usually resized to 224×224 to adapt to a pre-trained model.

### 4.4. Overall Evaluation

In reference to Section 3.2 and Section 3.3, we make an overall evaluation of all of the privacy-preserving DNNs here. ELE-AdaptNet can reduce the influence of block-wise encryption, but the degradation in accuracy and the increment in computation cost are still unacceptable, especially for large images. ViT-Enc with the ViT-B model had the highest performance on the CIFAR-10 dataset, but it was not robust against the ciphertext-only attack. In contrast, our proposed method not only achieved competitive performance on the CIFAR-10 and ImageNet datasets but also avoided a tremendous increment in computation cost. Furthermore, it was robust against all state-of-the-art ciphertext-only attacks. As a result, it is the best choice among these privacy-preserving methods in consideration of the requirements mentioned in Section 2.4.

## 5. Conclusions

In this paper, we proposed a novel privacy-preserving image classification method that uses ConvMixer with an adaptive permutation matrix and block-wise scrambled image encryption. The proposed method did not increase the computation cost too much compared with a model trained on plain images. In an experiment, the proposed method was demonstrated to outperform conventional methods in terms of classification accuracy, computation cost, and robustness against attack methods.

## Figures and Tables

**Table 1:** Classification accuracy (%) on CIFAR-10 dataset and computation cost of proposed and conventional privacy-preserving image classification methods. (✓) denotes “Strong”, and (✗) denotes “Weak”.

Encryption	Network	Image Size (Block-Size)	Accuracy (%)	# Parameters ≈($\times 10^{6})$	# FLOPs ≈($\times 10^{9})$	Security
LE [13,14]	Shakedrop ${}^{†}$	32(4)	94.49	29.31	4.73	✗
EtC [11,14]	Shakedrop ${}^{†}$	32(4)	89.09	29.31	4.73	✓
ELE [14]	Shakedrop ${}^{†}$	32(4)	83.06	29.31	4.73	✓
PE [26]	ResNet18	32(-)	91.33	11.18	0.04	✗
ViT-Enc [34]	ViT-B	224(16)	96.64	85.81	17.58	✗
Proposed	ConvMixer-512/16	224(16)	89.14	5.31	0.91	✓
Proposed	ConvMixer-512/16 ${}^{‡}$	224(16)	92.65	5.35	0.93	✓
Plain	ShakeDrop	32(-)	96.70	28.49	4.73	-
Plain	ViT-B	224(-)	99.11	85.81	17.58	-
Plain	ConvMixer-512/16	224(-)	96.80	5.31	0.91	-

${}^{†}$ Shakedrop with an ELE-AdaptNet. ${}^{‡}$ ConvMixer with an adaptive permutation matrix (proposed).

**Table 2:** Classification accuracy (%) on ImageNet of proposed privacy-preserving image classification method.

Encryption	Network	Image Size (Block-Size)	Accuracy (%)	# Parameters ≈($\times 10^{6})$	# FLOPs ≈($\times 10^{9})$
Proposed	ConvMixer-1024/20 ${}^{‡}$	224(16)	63.72	24.45	5.61
Plain	ConvMixer-1024/20	224(-)	76.94	24.38	5.55

${}^{‡}$ ConvMixer with an adaptive permutation matrix (proposed).

## Data Availability

The datasets used in this paper are publicly available at https://www.cs.toronto.edu/~kriz/cifar.html, accessed on 22 March 2023 and https://www.image-net.org/, accessed on 22 March 2023.
